# Relationship between IL-6 and COVID-19: to be considered during treatment

**DOI:** 10.2217/fvl-2020-0168

**Published:** 2020-12-24

**Authors:** Hafize Seda Vatansever, Eda Becer

**Affiliations:** 1 ^1^Department of Histology & Embryology, Faculty of Medicine, Manisa Celal Bayar University, Manisa, Turkey; 2 ^2^DESAM Institute, Near East University, Nicosia, Mersin 10 Turkey; 3 ^3^Department of Biochemistry, Faculty of Pharmacy, Near East University, Nicosia, Mersin 10 Turkey

**Keywords:** COVID-19, cytokine, cytokine storm, IL-6, SARS-CoV-2

## Abstract

The detection and control of pro-inflammatory response is crucial in the early stages of viral infection. Coronavirus disease 2019 (COVID-19) is an emerging viral disease of global concern and optimal treatment has yet to be determined. Unknown response of treatment of COVID-19 is important during patient monitoring. IL-6 is one of the key cytokines after activated macrophages. Therefore, control of systemic IL-6 levels in SARS-CoV-2 infected patients may be a parameter for COVID-19 disease. This review is focused on the induction of IL-6 after viral infections as a target molecule for monitoring cellular response.

IL-6 is a cytokine which controls the immune response in addition to cell proliferation and differentiation. Human IL-6 is a gene mapped in chromosome 7p21 and is comprised of 212 amino acids, including a 28-amino-acid signal peptide. IL-6 is a cytokine which has both pro- and anti-inflammatory properties. Secretion of IL-6 is also observed in atherosclerosis, Alzheimer’s, systemic lupus erythematosus, multiple myeloma, rheumatoid arthritis (RA), autoimmune deficiency disease, chronic inflammatory disease and different types of cancer etc. Therefore, the control of the IL-6 secretion is particularly important during disease, especially their activation after immune response activation [1,2].

## IL-6 & secretion from cells

IL-6 is secreted from several type of cells such as T cells, macrophages, endothelial cells, fibroblasts and monocytes [3]. The targets of the IL-6 are B cells, T cells, basophils, eosinophils and neutrophils. The functions of IL-6 on B cells are differentiation of the B cells as well as IgM, IgE and IgA production. In addition, IL-6 also controls the activation, differentiation and survival of the T cells. IL trigger the activation of leukocytes. Therefore, after infection, cytokine storm causes T and B cell activation and differentiation. Increased levels of IL-6 are demonstrated in low prognoses or metastatic cancers. IL-6 secretion causes antibody production from B cells and it enhances auto-antibody hypergammaglobulinemia. In addition, IL-6 causes auto-immunity, chronic inflammation and either CD4 positive T-cell activation, which triggers Th17 differentiation, or CD4 positive T-cell inhibition, which inhibits Treg differentiation [4].

After IL-6 activation, acute phase proteins are secreted in the initial stage of inflammation. These proteins are C-reactive protein (CRP), serum amyloid A (SAA), fibrinogen, haptoglobin and a1-antichymotrypsin, which are secreted from the liver. In addition, reductions of fibronectin, albumin and transferrin are also observed after IL-6 activation. Therefore, changes in the hepatocytes protein levels cause amyloid A amyloidosis, cardiovascular incurs, edema etc. Secretion of IL-6 induces hepcidin production as it blocks the activation of the iron transporter and causes hypoferremia and anemia associated with chronic inflammation. In an unexpected situation, if IL-6 reaches the bone marrow, it triggers megakaryocyte maturation and platelet formation. CD8 T cell differentiation also occurs after IL-6 activation. Other than these effects, collagen from dermal fibroblast, RANKL and VEGF from synovial fibroblast are also detected after IL-6 secretion. Therefore, fibrosis, angiogenesis and osteoporosis are expected effects of IL-6 secretion [5].

## Viral infection & IL-6

After viral infection, viral products also enhance the transcription or translation of IL-6 from cells such as fibroblast, mesenchymal, endothelial and many other cells. Therefore, the control of IL-6 synthesis and secretion may be important to inhibit its signal that affects cells. Suppression of IL-6 expression strategies can also be chosen to negatively regulate the IL-6 transcription [6,7]. The triggering of the IL-6 and the secretion of other cytokines after viral infection cause a fatal immune reaction to the hyperactivation of T cells. After cancer immunotherapy, the same cytokine storm is also observed because of T-cell activation and a boost in IL-6 secretion [8]. Therefore, pathological IL-6 secretion is thought to be the cause of the clinical symptoms after severe disease.

## Laboratory findings of COVID-19

Based on hospitalized patient data, substantial differences in laboratory findings were found in both deceased and recovered COVID-19 patients. In two reports where COVID-19 cases in Wuhan were investigated, white blood cell and neutrophil counts were significantly increased in correlation with disease severity. Also, COVID-19 patients had persistent and more severe lymphocytopenia and thrombocytopenia. A study reported that elevated neutrophils and reduced lymphocytes were also correlated with COVID-19 disease severity and death [9,10]. It can be suggested that a cellular level of immune deficiency state was related with poor prognosis.

The pathophysiology of SARS-CoV-2 with unusually high pathogenicity has not been exactly understood. In intensive care unit patients, pulmonary and extrapulmonary organ damage such as sepsis, heart failure, acute cardiac injury, acute kidney injury, shock, acidosis, alkalosis, acute liver injury may develop [11]. Recent studies have demonstrated that increased amounts of pro B type natriuretic peptide (NT-proBNP), creatine kinase-MB (CK-MB), high-sensitivity troponin-I (Hs-TnI), CRP, D-dimer and prothrombin were associated with inflammation-related cardiac problems. Additionally, these parameters were also reported as risk factors of systematic inflammation related cardiac injury and were also correlated with the risk of in-hospital death [10,12–14]. Furthermore, acute cardiac injury and heart failure may not be major fatality risk factors of COVID-19, but they could be co-existing disease outcomes of the acute respiratory distress syndrome and respiratory failure regardless of previous cardiovascular disease history.

Common laboratory abnormalities in COVID-19 patients included impaired liver and kidney function test parameters. Most patients had high concentrations of alanine aminotransferase, aspartate aminotransferase, total bilirubin, lactate dehydrogenase, alkaline phosphatase and γ-glutamyl transpeptidase and they were markedly higher in deceased patients compared with recovered patients. On the other hand, hypoalbuminaemia was shown in COVID-19 patients. Concentrations of blood urea nitrogen, ferritin, potassium and triglycerides were significantly increased in all COVID-19 patients, but were higher in deceased patients than in recovered patients [9,12]. Interestingly, a study reported significantly lower levels of thyroid stimulating hormone and free triiodothyronine in deceased patients than in recovered COVID-19 patients in China [9].

## Cytokines & COVID-19

Cytokine storm is an interesting point in COVID-19 patients. High levels of inflammatory cytokines were observed in COVID-19 patients with more severe disease and were associated with pulmonary inflammation, lung damage and multiple organ failure [15]. A previous study showed that increased levels of pro-inflammatory cytokines in serum such as IL1β, IL6, IL12, IFNγ, IP10 and MCP1 were related with pulmonary inflammation in SARS patients [16]. Additionally, Huang *et al.* reported high levels of IL1β, IFNγ, IP10 and MCP1 in intensive care unit COVID-19 patients, which is probably the reason for the activated T-helper-1 (Th1) cell response [12]. Moreover, Diao *et al.* found that the levels of TNF-α, IL-6 and IL-10 were correlated with the severity of COVID-19 [17].

IL-6 is an important cytokine whose production is related with various inflammatory diseases. Subjects with SARS-CoV-2 had high levels of IL-6 that were correlated with patient symptomatology including pulmonary inflammation and extensive lung damage [11]. Additionally, patients with SARS-CoV-2 infection had low levels of suppressor of cytokine signaling-3, which regulates and stimulates the negative feedback mechanism of IL-6 [18]. On the same line, another study reported that IL-6 levels were higher in severe COVID-19 patients and this may be used as one of the bases for predicting the transition from mild to severe infection [19]. In particular, Diao *et al.* showed that COVID-19 patients in intensive care had lower CD8^+^ T cell counts and their total CD4^+^ and CD8^+^ T cell counts were also negatively correlated with TNF-α and IL-6 concentrations [17]. In addition, recent studies showed that higher level of IL-6, CRP and also IL-10 were more significant rather than other cytokines in critical group of COVID-19 patients [20,21]. It can be suggested that immune dysregulation is a highly important point and therapeutic target for COVID-19 patients. The reasons for the large scale of the inflammatory cytokines are not clear, but it could play a crucial role in cell apoptosis associated with organ damage.

## Potential treatment methods of COVID-19

COVID-19 associated infections have been well described and include cytokine storm, inflammation, pathologic coagulation, endothelial dysfunction, kidney and heart problems [13]. However, there is no specific target antiviral treatment and no vaccine is currently available for COVID-19. The COVID-19 treatments include antiviral drugs treatments, steroids, plasma from recovered patients and mesenchymal stem cell transplantation.

Among the therapeutic strategies, no antiviral treatments have been approved but some molecules have been proposed such as chloroquine, baricitinib and hydroxychloroquine. Also, tocilizumab and anti-TNF-α are used for preventing cytokine storm. Chloroquine and hydroxychloroquine are 4-aminoquinolines and are used to treat malaria, lupus erythematosus and RA. A study reported that chloroquine and hydroxychloroquine inhibit SARS-CoV-2 *in vitro* [22]. Studies have also reported that they have broad-spectrum antiviral activity, particularly against SARS, and have recently been included in the Chinese and Italian guidelines for the treatment of COVID-19. Chloroquine increases the endosomal pH necessary for viral cell fusion and it also glycosylates ACE2 receptors and inhibits viral entry into cells [13,14,23]. On the other hand, Baricitinib is an another drug used to treat RA. It is a JAK inhibitor and can limit both cytokine production and inflammatory responses. Therefore, it may be useful in the prevention of the COVID-19-related cytokine storm and entry into cells [24,25].

IL-6 is one of the key mediators of inflammation and viral cytokine storm in COVID-19 patients [26]. Some studies have reported that the humanized monoclonal antibody against IL-6 receptors, tocilizumab, can be used in COVID-19 treatment based on its cytokine storm blocking property [27]. A recent Chinese retrospective study showed that tocilizumab improved fever, CRP levels and hypoxemia without leading to any significant adverse reactions in 21 severe COVID-19 patients [28]. According to the Italian guidelines, tocilizumab can only be used for COVID-19 patients who are at the end of the high viral load phase, with interstitial pneumonia, heavy respiratory insufficiency and high lL-6 and/or D-dimer/CRP/ferritin/fibrinogen levels [29]. TNF-α is an important mediator of cytokines and chemokines production and has a role in acute and chronic systemic inflammatory responses. The level of TNF-α was found to be high in COVID-19 patients and was correlated with disease severity [30]. In COVID-19 patients, anti-TNF-α treatment may be a potential choice and also a randomized and controlled adalimumab trial has been conducted [31]. Additionally, corticosteroids and immunosuppressants may be used for cytokine storm and is modulated systemic in COVID-19 patients. However, recent studies have shown that the use of hydrocortisone was related with a higher plasma SARS-CoV-2 viral load and delayed viral clearance in COVID-19 patients [32].

Scientists are working on novel therapies against the SARS-CoV-2 virus. Blood purification therapies are not novel methods but they can be used for COVID-19 treatment. A high level of antibodies has been found against the pathogen in recovered patients’ blood. These antibodies, immunoglobulin (Ig), are produced by B lymphocytes and also recognize and fight with molecules that are produced by the pathogens [33]. Ma *et al.* reported that that blood purification therapy was effective in COVID-19 patients [34]. In particular, blood purification therapy showed benefits in terms of the cytokine storm, via decreasing IL-6 and CPR levels in COVID-19 patients. On the other hand, mesenchymal stem cell transplantation is a novel treatment method for COVID-19. Mesenchymal stem cells have natural immunity to SARS-CoV-2 through ACE2- and TMPRSS2-properties and they also secrete anti-inflammatory factors to inhibit the cytokine storm. Leng *et al.* reported that the intravenous transplantation of mesenchymal stem cells was an effective and safe treatment for COVID-19 patients with pneumonia and a severe condition [35]. Mesenchymal stem cells can be accumulated in the lung and may promote endogenous repair via improving the pulmonary microenvironment. It can be suggested that blood purification therapies and mesenchymal stem cell transplantation are potentially powerful methods for COVID-19 treatment.

A systemic upregulation of IL-6 during the acute phase of viral infection shows the relationship between IL-6 levels and virus virulence. IL-6 is a pleotropic cytokine produced in response to tissue damage and infections [5]. In addition, elevated circulating levels of IL-6, TNF-α and MCP-1 were observed in virus infected human patients compared with virus negative groups [36]. Because the IL-6 secretion produced from multiple cell types starts the JAK/STAT3 activation pathway, it promotes several transcription factors associated with cellular signaling processes. IL-6 is considered to be one of the most important cytokines during an infection because it controls the differentiation of monocytes into macrophages, increases B-cell IgG production and also promotes the Th2 response by inhibiting Th1 polarization [37]. Production of IL-6 has been associated with both pro- and anti-inflammatory effects; therefore, further studies are needed to explain the IL-6 mediated cellular response during viral infections, especially COVID-19.

## Conclusion

The important role of IL-6 in host defense should always be considered in clinical practice. During COVID-19 treatments, several algorithms are used for patients who have several clinical symptoms. However, the responses of the treatment for cytokine storm, especially IL-6 production, are still unknown. Elevated systemic IL-6 levels according to COVID-19 severity should be important for determination of higher risk of disease deterioration. Therefore, monitoring of the IL-6 or targeting treatment of the IL-6 for COVID-19 positive patients may be a new target for effective treatment.

## Future perspective

SARS-CoV-2 was recognized in Wuhan and causes severe respiratory illness in humans, called COVID-19. A large number of vaccines and drugs are under investigation against COVID-19. Some of the vaccines showed promising results. New vaccines will become possible to create an immunity against SARS-CoV-2 and will be available in the market in future months. Another important point is that COVID-19 causes pulmonary and extrapulmonary organ damage which is mainly related to cytokine storm. IL-6 is the key molecule of cytokine storm, therefore IL-6 may be a target for drug development.

**Executive summary**

- IL-6 is an important cytokine with pleiotropic functions such as metabolic regulation to inflammation, auto-immunity and acute-phase response.
- COVID-19 patients had high levels of IL-6 that were associated with pulmonary inflammation and extensive lung damage.
- COVID-19 infection has an aggressive inflammatory response with a large amount of pro-inflammatory cytokines, known as the ‘cytokine storm’.
- Acute increase in circulating levels of pro-inflammatory cytokines including IL-6, IL-1, TNF-α and interferon is the reason for the cytokine storm.
- IL-6 may be a therapeutic target for inhibiting the cytokine storm and cytokine storm-associated organ damage.
